# Impact of *Wisteria floribunda* Agglutinin-Positive Mac-2-Binding Protein in Patients with Hepatitis C Virus-Related Compensated Liver Cirrhosis

**DOI:** 10.3390/ijms17091500

**Published:** 2016-09-12

**Authors:** Kunihiro Hasegawa, Ryo Takata, Hiroki Nishikawa, Hirayuki Enomoto, Akio Ishii, Yoshinori Iwata, Yuho Miyamoto, Noriko Ishii, Yukihisa Yuri, Chikage Nakano, Takashi Nishimura, Kazunori Yoh, Nobuhiro Aizawa, Yoshiyuki Sakai, Naoto Ikeda, Tomoyuki Takashima, Hiroko Iijima, Shuhei Nishiguchi

**Affiliations:** Division of Hepatobiliary and Pancreatic disease, Department of Internal Medicine, Hyogo College of Medicine, Hyogo 663-8501, Japan; hiro.red1230@gmail.com (K.H.); chano_chano_rt@yahoo.co.jp (R.T.); nishikawa_6392@yahoo.co.jp (H.N.); akio0010@yahoo.co.jp (A.I.); yo-iwata@hyo-med.ac.jp (Y.I.); yuho.0818.1989@gmail.com (Y.M.); ishinori1985@yahoo.co.jp (N.I.); gyma27ijo04td@gmail.com (Y.Y.); chikage@hyo-med.ac.jp (C.N.); tk-nishimura@hyo-med.ac.jp (T.N.); mm2wintwin@ybb.ne.jp (K.Y.); nobu23hiro@yahoo.co.jp (N.A.); sakai429@hyo-med.ac.jp (Y.S.); nikeneko@hyo-med.ac.jp (N.I.); tomo0204@yahoo.co.jp (T.T.); hiroko-i@hyo-med.ac.jp (H.I.); nishiguc@hyo-med.ac.jp (S.N.)

**Keywords:** hepatitis C virus, liver cirrhosis, hepatocellular carcinoma, *Wisteria floribunda* agglutinin-positive Mac-2-binding protein (WFA^+^-M2BP), survival, time-dependent receiver operating characteristics (ROC) analysis

## Abstract

We aimed to examine the effect of *Wisteria floribunda* agglutinin-positive Mac-2-binding protein (WFA^+^-M2BP) level on survival comparing with other laboratory liver fibrosis markers in hepatitis C virus (HCV)-related compensated liver cirrhosis (LC) (*n* = 165). For assessing prognostic performance of continuous fibrosis markers, we adapted time-dependent receiver operating characteristics (ROC) curves for clinical outcome. In time-dependent ROC analysis, annual area under the ROCs (AUROCs) were plotted. We also calculated the total sum of AUROCs in all time-points (TAAT score) in each fibrosis marker. WFA^+^-M2BP value ranged from 0.66 cutoff index (COI) to 19.95 COI (median value, 5.29 COI). Using ROC analysis for survival, the optimal cutoff point for WFA^+^-M2BP was 6.15 COI (AUROC = 0.79348, sensitivity = 80.0%, specificity = 74.78%). The cumulative five-year survival rate in patients with WFA^+^-M2BP ≥ 6.15 COI (*n* = 69) was 43.99%, while that in patients with WFA^+^-M2BP < 6.15 COI (*n* = 96) was 88.40% (*p* < 0.0001). In the multivariate analysis, absence of hepatocellular carcinoma (*p* = 0.0008), WFA^+^-M2BP < 6.15 COI (*p* = 0.0132), achievement of sustained virological response (*p* < 0.0001) and des-γ-carboxy prothrombin < 41 mAU/mL (*p* = 0.0018) were significant favorable predictors linked to survival. In time-dependent ROC analysis in all cases, WFA^+^-M2BP had the highest TAAT score among liver fibrosis markers. In conclusion, WFA^+^-M2BP can be a useful predictor in HCV-related compensated LC.

## 1. Introduction

Chronic hepatitis C (CHC) infection is a major cause of hepatocellular carcinoma (HCC) [1,2,3]. CHC also constitute the major etiologies of liver cirrhosis (LC) worldwide [1,2,3,4]. In our country, high annual HCC incidence rate in hepatitis C virus (HCV)-related LC patients had been reported (7%–8% per year) [5,6]. Clinical outcome in cirrhotic subjects is highly variable and can be influenced by several factors, such as liver disease etiology, severity of liver disease and presence of complications [4,7]. In decompensated LC patients with ascites or encephalopathy, survival decreases to one or two years [7,8,9].

On the other hand, the advent of direct-acting antivirals (DAAs) has spurred a revolution for CHC therapy with sustained viral response (SVR) rates exceeding 90% in the daily clinical practice [10]. The introduction of these highly effective DAAs for CHC patients is also expected to reduce the incidence of HCV-related HCC. However, the achievement of an SVR does not indicate that it completely eliminates the risk for HCC development, particularly when the subjects have already been complicated with LC [2,3,10,11,12].

Recently, Japanese investigators have established a novel liver fibrosis marker (*Wisteria floribunda* agglutinin-positive Mac-2-binding protein (WFA^+^-M2BP)), which is a glycobiomarker associated with CHC-related liver fibrosis with a unique fibrosis-related glycoalteration [13,14,15]. In addition, results for WFA^+^-M2BP can be obtained easily [13,14,15]. Thereafter, several investigators validated the usefulness of WFA^+^-M2BP on the liver fibrosis or clinical outcomes in various etiologies of chronic liver diseases (CLDs) [16,17,18,19,20,21,22,23,24,25]. Thus, clinical evidences with regard to WFA^+^-M2BP on liver fibrosis in CLDs have been accumulated recently.

However, to the best of our knowledge, there are no available data with regard to the effect of WFA^+^-M2BP level on survival in HCV-related compensated LC patients. As mentioned above, because clinical outcome in LC patients is highly variable, this investigation may be clinically important. The goal of our study is therefore to examine the effect of WFA^+^-M2BP level on survival comparing with other laboratory liver fibrosis markers in patients with HCV-related compensated LC. Moreover, in order to further assess prognostic performance of continuous liver fibrosis markers, we adapted time-dependent receiver operating characteristics (ROC) analysis for clinical outcome based on previous reports [26,27].

## 2. Results

### 2.1. Baseline Characteristics

The baseline characteristics in the current analysis are presented in Table 1 (*n* = 165). There are 93 males and 72 females with the median age of 67 years. For the entire cohort, the WFA^+^-M2BP value ranged from 0.66 cutoff index (COI) to 19.95 COI (median value, 5.29 COI). Sixty-eight out of 165 patients (41.2%) had HCC on radiological findings, which included stage I in 22, stage II in 26, stage III in 10 and stage IV in 10. The median observation period in the present analysis was 3.852 years (range: 0.219–9.241 years). As for HCV genotype and HCV viral load, genotype 1 (84.8% (140/165)) and high viral load as defined by HCV-RNA ≥ 5 log copies/mL (87.3% (144/165)) were in the majority at study entry. During follow-up period, SVR was achieved in 68 patients (41.2%) by antiviral therapies. Of these, 34 patients were treated with interferon-based antiviral therapies and the remaining 34 patients were treated with interferon-free antiviral therapies.

### 2.2. Cumulative Overall Survival Stratified by WFA^+^-M2BP for the Entire Cohort (n = 165)

**Figure 1:**
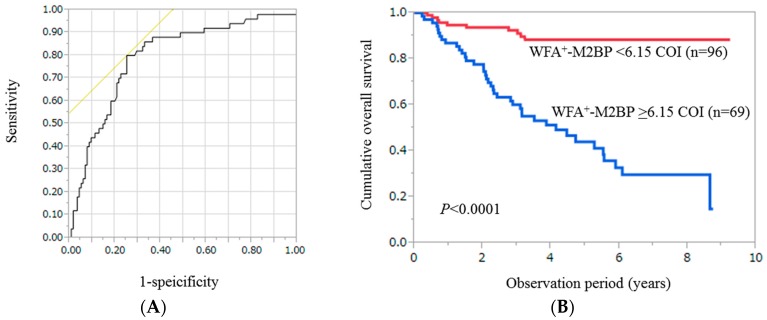
(**A**) Receiver operating characteristics (ROC) curves of *Wisteria floribunda* agglutinin-positive Mac-2-binding protein (WFA^+^-M2BP) for survival in all cases (*n* = 165). The optimal cutoff point for WFA^+^-M2BP level was 6.15 cutoff index (COI) (annual area under the ROC (AUROC) = 0.79348, sensitivity = 80.0%, specificity = 74.78%); (**B**) Cumulative overall survival stratified by WFA^+^-M2BP in all cases. The cumulative one-, three- and five-year survival rates were: 86.96%, 60.14% and 43.99%, respectively, in patients with WFA^+^-M2BP ≥ 6.15 COI (*n* = 69), while 94.76%, 92.41% and 88.40%, respectively, in patients with WFA^+^-M2BP < 6.15 COI (*n* = 96) (*p* < 0.0001).

Using ROC analysis, the optimal cutoff point for WFA^+^-M2BP level was 6.15 COI (AUROC = 0.79348, sensitivity = 80.0%, specificity = 74.78%) (Figure 1A). For the entire cohort, the cumulative one-, three- and five-year survival rates in patients with WFA^+^-M2BP ≥ 6.15 COI (*n* = 69) were 86.96%, 60.14% and 43.99%, respectively, while those in patients with WFA^+^-M2BP < 6.15 COI (*n* = 96) were 94.76%, 92.41% and 88.40%, respectively (*p* < 0.0001) (Figure 1B).

### 2.3. Cumulative Overall Survival Stratified by WFA^+^-M2BP for Patients with HCC (n = 68)

**Figure 2:**
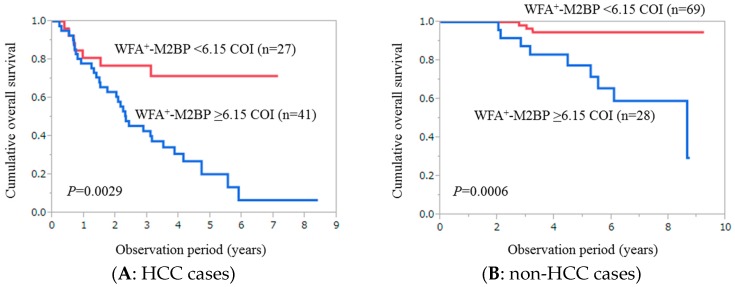
(**A**) Cumulative overall survival stratified by WFA^+^-M2BP for patients with hepatocellular carcinoma (HCC) (*n* = 68). The cumulative one-, three- and five-year survival rates were 78.05%, 42.8% and 20.27%, respectively, in patients with WFA^+^-M2BP ≥ 6.15 COI (*n* = 41), while 81.02%, 76.97% and 71.47%, respectively, in patients with WFA^+^-M2BP < 6.15 COI (*n* = 27) (*p* = 0.0026); (**B**) Cumulative overall survival stratified by WFA^+^-M2BP for patients without HCC (*n* = 97). The cumulative one-, three- and five-year survival rates were 100%, 87.5% and 77.58%, respectively, in patients with WFA^+^-M2BP ≥ 6.15 COI (*n* = 28), while 100%, 98.31% and 94.76%, respectively, in patients with WFA^+^-M2BP < 6.15 COI (*n* = 69) (*p* = 0.0006).

For HCC patients, the cumulative one-, three- and five-year survival rates in patients with WFA^+^-M2BP ≥ 6.15 COI (*n* = 41) were 78.05%, 42.8% and 20.27%, respectively, while those in patients with WFA^+^-M2BP < 6.15 COI (*n* = 27) were 81.02%, 76.97% and 71.47%, respectively (*p* = 0.0026) (Figure 2A).

### 2.4. Cumulative Overall Survival Stratified by WFA^+^-M2BP for Patients without HCC (n = 97)

For patients without HCC, the cumulative one-, three- and five-year survival rates in patients with WFA^+^-M2BP ≥ 6.15 COI (*n* = 28) were 100%, 87.5% and 77.58%, respectively, while those in patients with WFA^+^-M2BP < 6.15 COI (*n* = 69) were 100%, 98.31% and 94.76%, respectively (*p* = 0.0006) (Figure 2B).

### 2.5. Comparison of WFA^+^-M2BP Level in Patients with and without HCC

**Figure 3:**
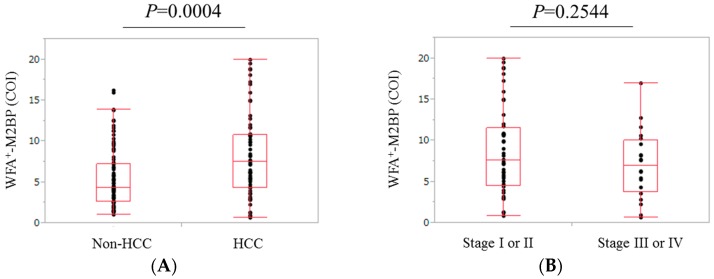
(**A**) Comparison of WFA^+^-M2BP level in patients with and without HCC (*p* = 0.0004); (**B**) Comparison of WFA^+^-M2BP level in patients with stage I or II HCC and stage III or IV HCC (*p* = 0.2554).

The median (range) WFA^+^-M2BP value in patients with HCC (7.50 COI (0.66–19.95 COI)) was significantly higher than that in patients without HCC (4.29 COI (1.06–16.24 COI)) (*p* = 0.0004) (Figure 3A).

### 2.6. Comparison of WFA^+^-M2BP Level in Patients with Stage I or II HCC and Those with Stage III or IV HCC

The difference of WFA^+^-M2BP levels in patients with stage I or II HCC (median: 7.57 COI, range: 0.8–17.95 COI) and those with stage III or IV HCC (median: 6.92 COI, range: 0.66–16.93 COI) did not reach significance (*p* = 0.2544) (Figure 3B).

### 2.7. Correlation between WFA^+^-M2BP Level and Tumor Markers for HCC Patients

**Figure 4:**
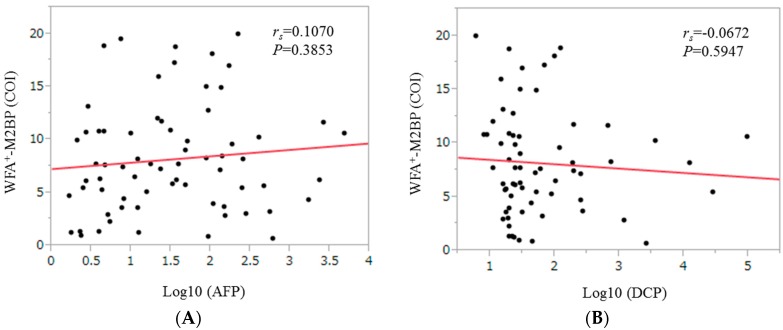
Correlation between WFA^+^-M2BP level and tumor markers (alpha-fetoprotein (AFP) (**A**) and des-γ-carboxy prothrombin (DCP) (**B**)) for HCC patients.

In patients with HCC, the WFA^+^-M2BP level did not significantly correlate with alpha-fetoprotein (AFP) (*r*_s_ = 0.1070, *p* = 0.3853) and des-γ-carboxy prothrombin (DCP) (*r*_s_ = −0.0672, *p* = 0.5947) (Figure 4).

### 2.8. Univariate and Multivariate Analyses of Factors Associated with Overall Survival for the Entire Cohort

Univariate analysis identified the following factors as significantly linked to overall survival (OS): age ≥ 71 years (*p* < 0.0001); presence of HCC (*p* < 0.0001); serum albumin ≥ 3.5 g/dL (*p* < 0.0001); prothrombin time ≥ 76% (*p* < 0.0001); platelet count ≥ 9.6 × 10^4^/mm^3^ (*p* = 0.0067); hyaluronic acid ≥ 265 ng/mL (*p* < 0.0001); WFA^+^-M2BP ≥ 6.15 COI (*p* < 0.0001); Fibrosis-4 (FIB-4) index ≥ 4.97755 (*p* = 0.0007); achievement of SVR during follow-up period (*p* < 0.0001); alkaline phosphatase ≥ 317 IU/L (*p* < 0.0001); AFP ≥ 10.8 ng/mL (*p* < 0.0001); and DCP ≥ 41 mAU/mL (*p* < 0.0001) (Table 2). The hazard ratios (HRs) and 95% confidence intervals calculated using multivariate analysis for the twelve factors with *p* value less than 0.05 in univariate analysis are shown in Table 2. Absence of HCC (*p* = 0.0008), WFA^+^-M2BP < 6.15 COI (*p* = 0.0132), achievement of SVR (*p* < 0.0001) and DCP < 41 mAU/mL (*p* = 0.0018) were revealed to be significant favorable predictors linked to OS.

### 2.9. Causes of Death

During the observation period, 50 patients (30.3%) died. The causes of death were HCC progression in 24 patients, liver failure in 19 patients and miscellaneous causes in seven patients.

### 2.10. Time-Dependent ROC Analyses for OS in all Cases

**Figure 5:**
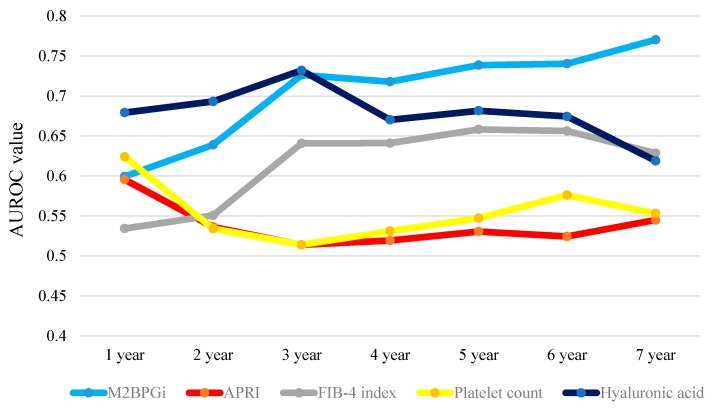
Time-dependent ROC analyses of five liver fibrosis markers (WFA^+^-M2BP, aspartate aminotransferase to platelet ration index (APRI), Fibrosis-4 (FIB-4) index, platelet count and hyaluronic acid) for overall survival (OS) in all cases. This figure presents the plots of annual AUROCs of five liver fibrosis markers. The total sum of AUROCs in all time-points (TAAT) score indicates total sum of AUROCs in all time-points in each liver fibrosis marker. TAAT score: WFA^+^-M2BP: 4.93226; APRI: 3.76484; FIB-4 index: 4.31010; platelet count: 3.87966 and hyaluronic acid: 4.74996.

Results for time-dependent ROC analyses at one-, two-, three-, four-, five-, six- and seven-year of WFA^+^-M2BP, APRI, FIB-4 index, platelet count and hyaluronic acid in all cases are presented in Table 3. The plots of annual AUROCs of five liver fibrosis markers as shown in Figure 5. The total sum of AUROCs in all time-points (TAAT score) in WFA^+^-M2BP was the highest among five liver fibrosis markers (TAAT score = 4.93226), followed by hyaluronic acid (TAAT score = 4.74996).

### 2.11. Time-Dependent ROC Analyses for OS in HCC Patients

**Figure 6:**
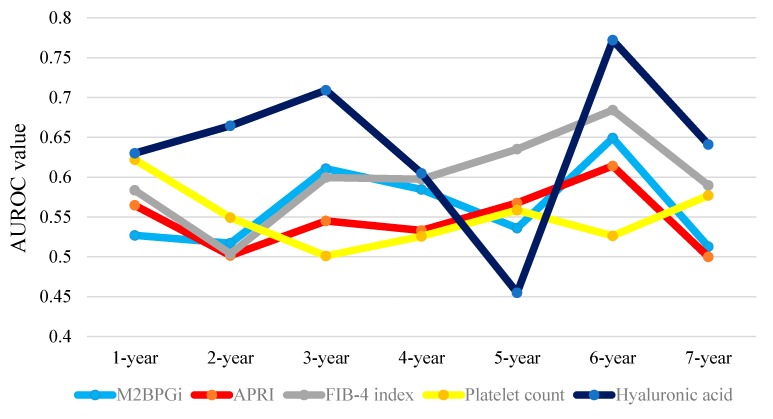
Time-dependent ROC analyses of five liver fibrosis markers (WFA^+^-M2BP, APRI, FIB-4 index, platelet count and hyaluronic acid) for OS in HCC cases. This figure presents the plots of annual AUROCs of five liver fibrosis markers. The TAAT score indicates total sum of AUROCs in all time-points in each liver fibrosis marker. TAAT score: WFA^+^-M2BP: 3.93734; APRI: 3.82610; FIB-4 index: 4.19352; platelet count: 3.85990 and hyaluronic acid: 4.47631.

Results for time-dependent ROC analyses at one-, two-, three-, four-, five-, six- and seven-year of WFA^+^-M2BP, APRI, FIB-4 index, platelet count and hyaluronic acid in HCC patients are shown in Table 3. The plots of annual AUROCs of five liver fibrosis markers are shown in Figure 6. The TAAT score in hyaluronic acid was the highest among five liver fibrosis markers (TAAT score = 4.47631), followed by FIB-4 index (TAAT score = 4.19352).

### 2.12. Time-Dependent ROC Analyses for OS in Non-HCC Patients

**Figure 7:**
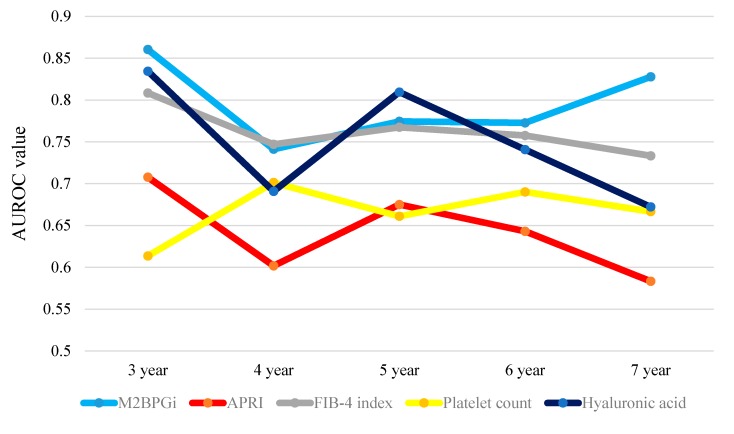
Time-dependent ROC analyses of five liver fibrosis markers (WFA^+^-M2BP, APRI, FIB-4 index, platelet count and hyaluronic acid) for OS in non-HCC cases. This figure presents the plots of annual AUROCs of five liver fibrosis markers. The TAAT score indicates total sum of AUROCs in all time-points in each liver fibrosis marker. TAAT score: WFA^+^-M2BP: 3.97663; APRI: 3.21116; FIB-4 index: 3.81393, platelet count: 3.33302 and hyaluronic acid: 3.74777.

Results for time-dependent ROC analyses at three-, four-, five-, six- and seven-year of WFA^+^-M2BP, aspartate aminotransferase to platelet ration index (APRI), FIB-4 index, platelet count and hyaluronic acid in non-HCC patients are shown in Table 3. The plots of annual AUROCs of five liver fibrosis markers are shown in Figure 7. The one- and two- year cumulative survival rate were both 100% in non-HCC patients and thus AUROCs in one- and two-year were not available. The TAAT score in WFA^+^-M2BP was the highest among five liver fibrosis markers (TAAT score = 3.97663), followed by FIB-4 index (TAAT score = 3.81393).

### 2.13. Comparison of the Proportion of A3 for Patients in Whom LC Was Diagnosed by Liver Biopsy According to WFA^+^-M2BP (n = 113)

As for liver inflammation stage (A stage) in histological findings, in patients in whom LC was diagnosed by liver biopsy (*n* = 113), 21 patients had A1 stage, 79 had A2 stage and 13 had A3 stage. The proportion of A3 in patients with WFA^+^-M2BP ≥ 6.15 COI was significantly higher than that in patients with WFA^+^-M2BP < 6.15 COI (20.51% (8/39) vs. 6.76% (5/74), *p* = 0.0293).

## 3. Discussion

HCV-related LC involves a highly heterogeneous condition with a broad spectrum of clinical characteristics, ranging from compensated stage to decompensated stage, each of which is characterized by a different prognosis [7,8,9]. It is obvious that decompensated LC patients had worse prognosis. We therefore conducted this study in limited patients with compensated LC. To our knowledge, this is the first report regarding the effect of WFA^+^-M2BP on clinical outcomes in HCV-related compensated LC patients. Furthermore, outcomes for CLDs are usually time-dependent. However, few studies have analyzed clinical data using time-dependent ROC analysis [26,27]. Thus, for detailed examination of the effect of WFA^+^-M2BP on clinical outcomes, we used both multivariate analysis and time-dependent ROC analysis, which took time-dependence into account [26,27].

In our results, WFA^+^-M2BP level ≥ 6.15 COI as determined by ROC analysis for survival was an independent adverse predictor linked to OS and for the entire cohort and non-HCC patients, it had the highest TAAT scores among liver fibrosis markers. In addition, cumulative survival rates were well stratified by WFA^+^-M2BP level irrespective of presence or absence of HCC. These results demonstrated that WFA^+^-M2BP can be helpful for predicting clinical outcome in HCV-related compensated LC. Our baseline data of WFA^+^-M2BP (range: 0.66–19.95 COI) may reflect the fact that HCV-related compensated LC patients had heterogeneous patients population. The insignificant correlation between WFA^+^-M2BP level and AFP or DCP values indicate that WFA^+^-M2BP could not be an alternative tumor marker to AFP or DCP.

In our previous study, we reported that WFA^+^-M2BP correlated with not only the degree of liver fibrosis but also the degree of liver inflammation activity [20,21,25]. Indeed, the proportion of A3 in patients with WFA^+^-M2BP ≥ 6.15 COI was significantly higher than that in patients with WFA^+^-M2BP < 6.15 COI in this study (*p* = 0.0293), and this may be associated with worse clinical outcome in patients with higher WFA^+^-M2BP level [7,28]. The statistical significance between non-HCC and HCC patients in terms of WFA^+^-M2BP level can be also explained by liver inflammation, although the difference of the proportion of A3 in HCC and non-HCC patients with available liver biopsy data did not reach significance (*p* = 0.2299, data not shown).

In HCC patients, hyaluronic acid had the highest TAAT score among five liver fibrosis markers. In this population, the presence of HCC itself can affect prognosis. On the other hand, in our previous study, we reported that serum hyaluronic acid could predict protein-energy malnutrition (PEM) in HCV-related liver disease [29]. Especially in this patient population, sufficient management for PEM may be essential for ameliorating outcome since HCC therapy can accelerate progression of PEM.

Notably, an achievement of SVR during follow-up period was the strongest favorable predictor linked to OS with the HR of 23.833 (*p* < 0.0001) in our multivariate analysis. A recent meta-analysis demonstrated that CHC subjects with SVR have a substantially reduced risk for HCC incidence (HR: 0.1–0.25), liver-related mortality (HR: 0.03–0.2) and all-cause mortality (HR: 0.1–0.3) as compared with no antiviral treatment or treatment failure, which are in agreement with our current results [30]. In compensated LC patients, antiviral therapy can be strongly recommended for improving clinical outcomes.

We acknowledge several limitations in this study. First, since this longitudinal study had a retrospective nature, our results should be cautiously interpreted; Second, in 52 patients (31.5%), LC was diagnosed not by liver biopsy but radiological findings alone, leading to bias; Third, liver biopsy involves restriction being prone to errors in a statistical analysis arising from the unrepresentativeness of the sample taken for assessing the degree of liver fibrosis, also leading to bias; Fourth, there were several missing values in our analysis; Fifth, calculating TAAT score is not a well established assessment method for predicting survival and this is only a proposal. However, our current results demonstrated that WFA^+^-M2BP performed well as a prognostic marker in HCV-related compensated LC patients.

Finally, we concluded that WFA^+^-M2BP can be a useful predictor in patients with HCV-related compensated LC.

## 4. Patients and Methods

### 4.1. Patients

Between March 2007 and June 2015, a total of 165 individuals with HCV-related compensated LC with available stored serum samples were admitted to the Division of Hepatobiliary and Pancreatic disease, Department of Internal Medicine, Hyogo College of Medicine, Hyogo, Japan and they were subjected to this analysis. Compensated LC was defined as Child-Pugh A LC. Subjects with HCV-related liver disease are defined as those with HCV antibody positive and hepatitis B surface antigen negative. In patients who did not receive liver biopsy (*n* = 52), LC was diagnosed through radiological findings: clinical features suggestive of portal hypertension such as splenomegaly, varices, ascites and a shrunken or deformed and nodular liver as identified on computed tomography (CT) or ultrasonography (US) findings [7]. The primary endpoint in our study is overall survival (OS). Firstly, we examined the relationship between WFA^+^-M2BP level and baseline characteristics or clinical outcomes. Subsequently, we investigated variables linked to OS using univariate and multivariate analyses. Finally, we compared the effect of WFA^+^-M2BP level on survival with those of other liver fibrosis markers including APRI, FIB-4 index, platelet count and hyaluronic acid using time-dependent ROC analysis. In principle, in patients with serum albumin level ≤3.5 g/dL, branched chain amino acids were administered [7]. In patients without HCC or those with curative treatment for HCC, antiviral therapies including interferon-based therapies or interferon-free therapies were also considered [7].

The ethics committee of Hyogo college of medicine approved the current study protocol (approval number: 1831; date: 1 March 2016) and this study protocol adhered to all provisions of the Declaration of Helsinki.

### 4.2. Measurement of WFA^+^-M2BP and Calculation of ARPI and FIB-4 Index

We tested WFA^+^-M2BP level using stored serum sample and it was measured based on a lectin-Ab sandwich immunoassay using the fully automatic immunoanalyzer, HISCL-2000i (Sysmex Co., Hyogo, Japan) as reported previously [31,32]. We calculated APRI score as described elsewhere: aspartate aminotransferase (AST/upper limit of normal)/platelet count (expressed as platelets × 10^9^/L) × 100 [33]. We calculated FIB-4 index as described elsewhere: age (years) × AST (IU/L)/platelet count (×10^9^/L) ×√alanine aminotransferase (ALT) (IU/L) [34].

### 4.3. HCC Surveillance and HCC Therapy

Follow-up consisted of periodical evaluation by imaging studies including US, CT and/or magnetic resonance imaging for HCC incidence or HCC recurrence and periodical blood tests such as tumor markers. For cases with HCC, most adequate therapy for each case was selected according to Japanese guidelines through discussion with radiologists, surgeons and hepatologists [35,36,37].

### 4.4. Statistical Analysis

In continuous parameters, groups were compared by using Student’s *t*-test or Mann–Whitney *U* test, or Spearman’s rank correlation coefficient r_s_, as applicable. In categorical parameters, groups were compared by using Fisher’s exact tests or Pearson χ^2^ test, as applicable. In continuous parameters, we performed ROC curve analysis of survival for selection of the optimal cutoff value that is associated with maximal total value of specificity and sensitivity and we classified them into two groups using these cutoff values and treated them as nominal covariates. Kaplan–Meier curves were generated and compared by using the log-rank test. Parameters with *p* value less than 0.05 in the univariate analysis were entered into the multivariate analyses (Cox proportional hazard model). Furthermore, we analyzed time-dependent ROC curves of WFA^+^-M2BP, APRI, FIB-4 index, platelet count and hyaluronic acid for survival and compared between area under the ROCs (AUROCs) for these liver fibrosis markers in each time point (one-, two-, three-, four-, five-, six-, and seven-year) [26,27]. We also calculated the total sum of AUROCs in all time-points (TAAT score) in each liver fibrosis marker.

OS was calculated as the time interval from the date at which we obtained stored serum sample during hospitalization with the purpose of liver biopsy, HCC therapy or others until death (due to any cause) or the last follow-up visit. Data are presented as median (range) unless otherwise stated. We regarded variables with *p* value less than 0.05 as statistically significant variables. Statistical analysis was performed using JMP 11 (SAS Institute Inc., Cary, NC, USA).

## Figures and Tables

**Table 1:** Baseline characteristics (*n* = 165).

Variables	Number or Median Value (Range)
Age (years)	67 (23–93)
Gender, male/female	93/72
HCC, None/stage I/II/III/IV	97/22/26/10/10
Total bilirubin (mg/dL)	0.8 (0.3–2.0)
Serum albumin (g/dL)	3.7 (2.9–4.9)
Prothrombin time (%)	81 (58.8–115.6)
Platelet count (×10^4^/mm^3^)	10.8 (1.7–38.7)
Hyaluronic acid (ng/mL)	201 (22–2270)
WFA^+^-M2BP (cutoff index)	5.29 (0.66–19.95)
AST (IU/L)	52 (17–343)
ALT (IU/L)	46 (7–396)
ALP (IU/L)	282 (130–985)
GGT (IU/L)	44 (12–357)
AFP (ng/mL)	10.6 (1.2–4867)
DCP (mAU/mL) ^#^	24 (6–96900)
HCV genotype 1/2/unknown	140/23/2
HCV viral load ≥ 5 log IU/mL, yes/no	144/21
MELD score	3.7 (−5.0–23.7)

Data are expressed as number or median (range). HCC, hepatocellular carcinoma; WFA^+^-M2BP, *Wisteria floribunda* agglutinin-positive Mac-2-binding protein; AST, aspartate aminotransferase; ALT, alanine aminotransferase; ALP, alkaline phosphatase; GGT, gamma glutamyl transpeptidase; AFP, alpha-fetoprotein; DCP, des-γ-carboxy prothrombin; ^#^, missing data (*n* = 8); HCV, hepatitis C virus; MELD, model for end-stage liver disease.

**Table 2:** Univariate and multivariate of factors associated with overall survival.

Variables	Number	Univariate Analysis (*p* Value)	Multivariate Analysis		
			HR	95% CI	*p* Value
Age ≥ 71 years, yes/no	60/105	<0.0001	2.110	0.919–4.950	0.0783
Gender, male/female	93/72	0.2684			
Presence of HCC, yes/no	68/97	<0.0001	4.527	1.833–12.320	0.0008
Total bilirubin ≥ 0.8 mg/dL, yes/no	97/68	0.3132			
Serum albumin ≥ 3.5 g/dL, yes/no	122/43	<0.0001	0.910	0.439–1.885	0.7975
Prothrombin time ≥ 76%, yes/no	112/53	<0.0001	0.816	0.410–1.621	0.5611
Platelet count ≥ 9.6 × 10^4^/mm^3^, yes/no	116/49	0.0067	0.697	0.313–1.554	0.3761
Hyaluronic acid ≥ 265 ng/mL, yes/no	59/96	<0.0001	1.942	0.890–4.382	0.0964
WFA^+^-M2BP ≥ 6.15 COI, yes/no	69/96	<0.0001	2.870	1.242–6.940	0.0132
APRI ≥ 1.27615, yes/no	92/73	0.0552			
FIB-4 index ≥ 4.97755, yes/no	80/85	0.0007	1.649	0.681–4.336	0.2758
Achievement of an SVR, yes/no	68/97	<0.0001	23.833	4.579–439.545	<0.0001
AST ≥ 55 IU/L, yes/no	76/79	0.0595			
ALT ≥ 20 IU/L, yes/no	148/17	0.3304			
ALP ≥ 317 IU/L, yes/no	68/97	<0.0001	1.161	0.545–2.553	0.7022
GGT ≥ 58 IU/L, yes/no	49/116	0.1018			
AFP ≥ 10.8 ng/mL, yes/no	82/83	<0.0001	1.426	0.659–3.318	0.3768
DCP ≥ 41 mAU/mL, yes/no ^#^	37/120	<0.0001	3.543	1.602–7.976	0.0018
MELD score ≥ 5.1, yes/no	55/110	0.0006	1.471	0.760–2.874	0.2521

HCC, hepatocellular carcinoma; WFA^+^-M2BP, *Wisteria floribunda* agglutinin-positive Mac-2-binding protein; COI, cutoff index; APRI, aspartate aminotransferase to platelet ration index; SVR, sustained virological response; AST, aspartate aminotransferase; ALT, alanine aminotransferase; ALP, alkaline phosphatase; GGT, gamma glutamyl transpeptidase; AFP, alpha-fetoprotein; DCP, des-γ-carboxy prothrombin; MELD, model for end-stage liver disease; ^#^, missing data (*n* = 8); HR, hazard ratio; CI, confidence interval.

**Table 3:** Results for time-dependent receiver operating characteristics analysis.

**All Cases**	**1-Year**	**2-Year**	**3-Year**	**4-Year**	**5-Year**	**6-Year**	**7-Year**	**Total Value**
WFA^+^-M2BP	0.59942	0.63898	0.72643	0.71793	0.73864	0.74045	0.77041	4.93226
APRI	0.59556	0.53636	0.51389	0.51932	0.53050	0.52431	0.54490	3.76484
FIB-4 index	0.53427	0.55076	0.64085	0.64111	0.65829	0.65625	0.62857	4.31010
Platelet count	0.62403	0.53415	0.51403	0.53104	0.54725	0.57610	0.55306	3.87966
Hyaluronic acid	0.6793	0.69324	0.73218	0.67026	0.68162	0.67448	0.61888	4.74996
**HCC Cases**	**1-Year**	**2-Year**	**3-Year**	**4-Year**	**5-Year**	**6-Year**	**7-Year**	**Total Value**
WFA^+^-M2BP	0.52695	0.51717	0.61068	0.58456	0.53604	0.64912	0.51282	3.93734
APRI	0.56469	0.50166	0.54505	0.53309	0.56757	0.61404	0.50000	3.82610
FIB-4 index	0.58356	0.50388	0.59956	0.59743	0.63514	0.68421	0.58974	4.19352
Platelet count	0.62197	0.54928	0.50111	0.52574	0.55856	0.52632	0.57692	3.85990
Hyaluronic acid	0.63005	0.66445	0.70912	0.60478	0.45495	0.77193	0.64103	4.47631
**Non-HCC**	**1-Year**	**2-Year**	**3-Year**	**4-Year**	**5-Year**	**6-Year**	**7-Year**	**Total Value**
WFA^+^-M2BP	NA	NA	0.86039	0.74122	0.77451	0.77273	0.82778	3.97663
APRI	NA	NA	0.70779	0.60187	0.67507	0.64310	0.58333	3.21116
FIB-4 index	NA	NA	0.80844	0.74707	0.76751	0.75758	0.73333	3.81393
Platelet count	NA	NA	0.61364	0.70141	0.66106	0.69024	0.66667	3.33302
Hyaluronic acid	NA	NA	0.83442	0.69087	0.80952	0.74074	0.67222	3.74777

WFA^+^-M2BP, *Wisteria floribunda* agglutinin-positive Mac-2-binding protein; APRI, aspartate aminotransferase to platelet ration index; HCC, hepatocellular carcinoma; NA, not available.

## Abbreviations

CHC, chronic hepatitis C; HCC, hepatocellular carcinoma; LC, liver cirrhosis; HCV, hepatitis C virus; DAAs, direct acting antivirals; SVR, sustained virological response; WFA^+^-M2BP, *Wisteria floribunda* agglutinin-positive Mac-2-binding protein; CLDs, chronic liver diseases; ROC, receiver operating characteristics; CT, computed tomography; US, ultrasonography; OS, overall survival; APRI, aspartate aminotransferase to platelet ration index; AST, aspartate aminotransferase; ALT, alanine aminotransferase; AUROCs, area under the ROCs; TAAT, total sum of AUROCs in all time-points; COI, cutoff index; AFP, alpha-fetoprotein; DCP, des-γ-carboxy prothrombin; HR, hazard ratio.
